# Supplementary material for: Identification of Freezing-Responsive microRNAs and Their Targets in Chinese Jujube by Small RNA and Degradome Sequencing
Source: Int J Mol Sci. 2026 Sep 4;27(17):7912. doi: 10.3390/ijms27177912 (PMC13566890; doi:10.3390/ijms27177912)
Supplement: Supplementary file 1 [file ijms-27-07912-s001.zip › Supplementary Material S2/novel_miR_150.pdf]

Provisional ID	:	novel_miR_150
Score total	:	59.6
Score for star read(s)	:	3.9
Score for read counts	:	53.7
Score for mfe	:	0.4
Score for randfold	:	1.6
Score for cons. seed	:	
Total read count	:	117
Mature read count	:	112
Loop read count	:	2
Star read count	:	3

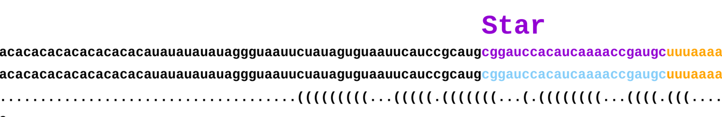

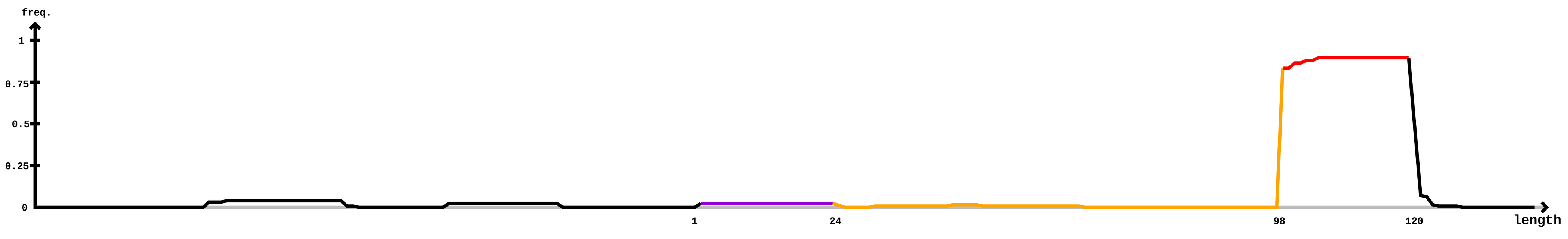

Star

Mature

```
5'- auauuaguuaugauauuaaagacuuaauuugauacuuguugacucaaacacacacacacacacacacauauauauauaggguaauucuauaguguaauucauccgcaugcggauccacaucaaaaccgaugcuuuaaaaaaaaaaaaaaagaucggcuuugcuuuuuguauguaaaagcaaaaccgauacuuuuaaaaaaaaaaaaacaucaacuuugguguaguacgcaugcagaugauccgcacuguagu -3' obs
    auauuaguuaugauauuaaagacuuaauuugauacuuguugacucaaacacacacacacacacacacauauauauauaggguaauucuauaguguaauucauccgcaugcggauccacaucaaaaccgaugcuuuaaaaaaaaaaaaaaagaucggcuuugcuuuuuguauguaaaagcaaaaccgauacuuuuaaaaaaaaaaaaacaucaacuuugguguaguacgcaugcagaugauccgcacuguagu     exp
```

read	reads	mm	sample
uuugauacuuguugacucaaaca	1	0	JCK-3
uuugauacuuguugacucaaaca	3	0	JCK-2
gauacuuguugacucaaacaca	1	0	LT-2
auauauauauagggGaauu	1	1	JCK-3
auauauauauagggGaauu	2	1	JCK-1
cggauccacaucaaaaccgaugU	2	1	JCK-3
cggauccacaucaaaaccgaugU	1	1	JCK-2
aaaaaaaaaaagaucUgc	1	1	LT-3
ucggcuuugcuuuGuguaugua	1	1	LT-2
aucaacuuugguguaguacgca	42	0	LCK-1
aucaacuuugguguaguacgca	60	0	LCK-2
auNaacuuugguguaguacgca	2	1	LCK-1
aucaacuuugguguaguacgcaug	1	0	LT-1
caacuuugguguaguacgcaugc	4	0	LT-2
acuuugguguaguacgcaugc	2	0	LT-1
uuugguguaguacgcaugca	1	0	LT-3
uuugguguaguacgcaugcagaug	1	0	LT-1
